# Moral judgments in online discourse are not biased by gender

**DOI:** 10.1038/s41598-025-08749-x

**Published:** 2025-07-01

**Authors:** Lorenzo Betti, Paolo Bajardi, Gianmarco De Francisci Morales

**Affiliations:** 1 ISI Foundation, 10126 Turin, Italy; 2 Department of Network and Data Science, Central European University, 1100 Vienna, Austria; 3 CENTAI, 10138 Turin, Italy

**Keywords:** Social norms, Moral judgment, Gender, Reddit, Computer science, Information technology

## Abstract

The interaction between social norms and gender roles prescribes gender-specific behaviors that influence moral judgments. While previous work has demonstrated the existence of gender-bias in judgments, these studies are mainly based on controlled experiments that may not reflect real-world decision-making processes. Here, we study how moral judgments are biased by the self-disclosed gender of the protagonist of a story. Using data from /r/AITA, a Reddit community with 17 million members who share first-hand experiences seeking community judgment on their behavior, we employ machine learning techniques to match stories describing similar situations that differ only by the protagonist’s gender. We find no direct causal effect of the protagonist’s self-disclosed gender on the received moral judgments, except for stories about “friendship and relationships”, where male protagonists receive more negative judgments. Our findings complement existing correlational studies and suggest that gender roles may exert greater influence in specific social contexts. These results have implications for understanding sociological constructs and highlight potential biases in data used to train large language models.

## Introduction

Social norms are the informal rules that govern behavior in groups and societies, and represent collective beliefs about which behavior is appropriate in a given situation. These norms are intimately connected to social roles—behavioral archetypes that define specific expectations for individuals within a social context^1,2^. Among these roles, gender roles stand out because they set expectations for men and women simply by virtue of who they are. How these gender-based expectations overlap with—and differ from—other social norms is still an active topic of research^3^. By prescribing gender-specific behaviors that become internalized, gender roles shape both how people act and how they render moral judgments—evaluations of right and wrong that elicit social approval or sanction^4^.

Investigating how social and gender roles influence moral judgments allows us to better understand human decision-making processes. Previous research offers mixed evidence about the role of demographic attributes in received moral judgments. On the one hand, variations of the trolley dilemma show no difference in blame assignment when only the protagonist’s gender is changed^5^. On the other hand, moral transgressors who are older (vs. younger) or female (vs. male) tend to receive comparatively milder judgments in fictitious stories^6,7^. However, most of this research relies on controlled experiments employing questionnaires or vignettes—short stories based on fictional or hypothetical scenarios designed to elicit moral judgments^8,9^. While this methodology proves effective in manipulating actors’ characteristics to investigate how they modulate moral judgments, the stories may not reflect the complexity of real-world situations, thus limiting the generalizability of the findings.

Social media provide a compelling data source to study gender bias in moral judgments at scale. Indeed, online communities aimed at social support serve as a natural experiment to study how self-disclosed demographic attributes influence moral judgments. In addition to enriching our understanding of human behavior, this setting allows for a better understanding of socio-technical systems, since moral judgments are mediated by online interactions.

The present study uses self-reported stories on Reddit to address our main research question: *“Does the declared gender of the protagonist of a story affect the moral judgment they receive?” * In particular, the subreddit /r/AmItheAsshole (/r/AITA) is one of several communities dedicated to discussing conflicts that arise in everyday life and expressing moral judgments on these situations. This collection of moral judgments is invaluable to understanding social norms. In particular, /r/AITA is particularly suitable for our study because it is relatively easy to collect demographic information of posters and community judgments. Indeed, protagonists often self-disclose their demographic attributes (i.e., age and gender) and the community expresses their judgment by using a defined set of tags, thus enabling the collection of protagonists’ demographics and the moral judgments they receive at scale. This information can then be used to study the interplay between demographics and social norms across a wide spectrum of contexts. We note that the study was not preregistered.

Other studies have investigated the relationship between demographics and moral judgment on Reddit, finding that male authors are more likely to be judged to deserve blame^10–13^. However, these works are correlational and may not account for factors that can bias the observed association. While they propose several hypotheses about the causal mechanisms behind this imbalance, they do not provide any concrete evidence for them. The present work addresses this gap: we design a causal observational study to understand the effect of the declared gender on the moral judgments received.

**Fig. 1:**
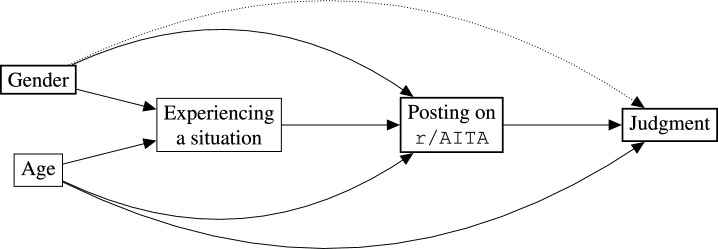
Causal graph encoding our hypotheses on the judgment mechanism in /r/AITA. Given a submission, we assume that the type of situation experienced (“*Experiencing a situation*”) is influenced by the gender and age of the protagonist (“*Gender*” and “*Age*”). Then, these three elements all affect the likelihood that the protagonist shares this situation on /r/AITA (“Posting on /r/AITA”). Finally, the moral judgment received (“*Judgment*”) is caused by the gender of the protagonist through two causal paths: directly, which reflects the first hypothesis, and mediated by the type of situation described in the submission, which refers to the second hypothesis. The dashed edge from “*Gender*” to “*Judgment*” is the causal effect we aim to measure.

Two competing possible explanations have been proposed for the observed disparity in negative judgments received by men and women, which are depicted in the causal graph of Figure 1. The first hypothesis is that judges are biased by the gender of the protagonist of the story because of gender stereotypes or homophilic effects^7,14^. Because agency is central to moral judgments^15^, men, who are stereotypically seen as more agentic than women, are more likely to be judged as perpetrators and receive blame. In contrast, women, who are stereotypically viewed as lower in agency, are more likely to be judged as patients^6^. This relationship between gender stereotypes and moral typecasting may contribute to moral judgment bias, whereby men are more frequently assigned blame. In this scenario, the gender of the protagonist would have a direct effect on the judgment received. If this hypothesis holds, one would expect a bias where male protagonists receive more negative judgments, as reported in prior observational^13^ and experimental studies^6^. The second hypothesis attributes the observed disparity in negative judgments to a disclosure bias, whereby male authors are more likely to share morally ambiguous situations and consequently receive negative judgments. Prior work shows that men are more comfortable with risky situations^16–18^, whereas women tend to use online communities as support groups^19–21^. Recent Reddit studies suggest this mechanism: female authors more often frame their experiences as events that happen to them^10,22^, which may lead to more lenient judgments toward them as a form of social support^11^. These gendered differences in what is posted create an indirect causal pathway that results in the observed disparity in negative judgments, mediated by the situation described. Therefore, if this hypothesis were true, controlling for the situations described would reveal no direct effect of gender on moral judgments. In sum, we aim to verify whether the situation is a partial or full mediator between gender and judgment.

To measure the direct effect of gender on judgment, our study design compares the judgment received by matched pairs of stories that describe similar situations but have protagonists of different genders. In that way, we can block the indirect causal path from gender to judgment and isolate the direct effect of interest. We estimate the causal effect of the protagonist’s gender (exposure) on the judgment received (outcome) by employing an approach based on propensity score matching^23^, which allows controlling for confounding variables. We enforce matching between similar stories authored by male and female users by computing semantic similarities between document embeddings derived from large language models. We also control for other factors such as the age of the protagonist and the general topic of discussion. We then manually evaluate the matched pairs of submissions by evaluating the situational similarity of the pairs.

Our results indicate no significant direct causal effect between the gender of the protagonist and the judgment of the community. When controlling for the situation described in the story, male protagonists are no more likely to receive a negative judgment than female ones. However, when disaggregating the results by topic, we find a small but significant negative bias towards male protagonists in only one of them: “friendship and relationships”. Possibly, the difference in this specific topic is due to social norms that are more connected to gender roles than in other contexts^10^.

The question tackled in this work has clear societal implications, as it sheds light on the interaction between fundamental sociological constructs. The fact that this bias might be present within an online social platform such as Reddit has additional relevance in an AI-focused era, as large language models use Reddit extensively as training data. If such causal bias were evident, then models trained on this data would be likely to reproduce and possibly amplify it further.

## Methods

### Data

/r/AITA is a subreddit where users submit real-life experiences to seek feedback on their actions from the community. Community members make comments under users’ submissions and eventually vote on the protagonist’s behavior by using judgment tags. These tags—*Not The Asshole* (NTA), *No Assholes Here* (NAH), *You’re The Asshole* (YTA), and *Everyone Sucks Here* (ESH)—are commonly used in the community and listed in the voting guidelines of the subreddit, explicitly assigning blame or absolution; we can therefore treat them as moral judgments rather than mere preferences^24^. In addition, community members can upvote comments they agree with (while downvotes are reserved for off-topic discussions or spam). The difference between upvotes and downvotes is shown in Reddit comments and is known as the *score* of the comment.

We collected all submissions posted on /r/AITA between 2014 and 2020, retrieving all the comments containing one of the judgment tags from the Pushshift Reddit data collection^25^. Since we are interested in the community judgment towards the protagonist of the submission, we merge YTA with the ESH tag, and the NTA with the NAH tag, as they convey the same judgment towards the protagonist. These new tags are named AH and N_AH respectively. After discarding content authored by bot accounts, the AITA dataset is composed of 250770 submissions and 6891476 comments (see Supplemental Material for additional details about the data collection).

### Topic detection

The submissions in the AITA dataset cover a wide range of subjects such as stories about parenting, work, or issues related to living with other people, just to mention a few. We train a Latent Dirichlet Allocation (LDA) topic model^26^ on all the submissions to estimate the probability that a submission belongs to each of $N_T=6$ topics. We use the LDA implementation of the Gensim library^27^. The number of topics is chosen by minimizing the perplexity on a held-out set of submissions in a 5-fold cross-validation setting (see Supplementary Fig. S1). Then, we assign each submission to the topic with the highest probability, provided that this probability is higher than the threshold 0.4. Submissions not meeting this threshold are assigned to a topic called “Other”, which includes submissions whose topic is either not well identified or a combination of the other topics. Supplementary Table S1 shows the 15 most relevant words for each topic according to the trained model. We interpret and give a title to each topic by looking at this table and reading the 5 submissions with the highest probability for each topic. We depict the distribution of submissions across topics in Supplementary Figure S4. Because narratives belonging to different topics may have different levels of moral relevance^28^, we report all main analyses both pooled and stratified by topic, so that any topic-specific variation in moral relevance is made explicit.

### Extraction demographics and judgments

To determine the community judgment for each submission we extract judgment tags from comments (see Supplemental Material for additional details). We then weight the judgment tags by the comment’s score (i.e., the difference between upvotes and downvotes) and assign the judgment corresponding to the tag with the largest weighted score. This approach considers all the comments that express a judgment, including all users who upvoted such comments. We discard submissions whose weight is lower than 10 to exclude submissions with few judgments.

To infer the author’s gender and age, we extract specific demographic tags commonly employed by users to provide more context to the community. For instance, “F26” denotes that the author identifies as a female aged 26, whereas “22 M” refers to a male aged 22. We use a regular expression to extract this information from the AITA dataset if such tags occur in the proximity of first-person singular pronouns, which indicates that the tags refer to the author of the submission. We identify the age and gender of the author in $15.6\%$ of the submissions. We also verify that topics have a uniform distribution of submissions with available demographics to confirm that the self-disclosure of gender does not depend on the topic (see Supplementary Fig. S2).

### Estimation of gender bias on judgment

We aim to determine whether self-disclosing a male gender leads to more negative moral judgments on /r/AITA than self-disclosing a female gender. In an ideal scenario, we would run a controlled experiment where participants judge the *same* submission where the gender of the author is assigned randomly. In such a way, the comparison between the judgments given to the submission where the author is a male against the ones where the author is a female would correspond to the effect of gender on judgment. Such an intervention, however, is not feasible on Reddit and we have to rely on observational data instead. Consequently, we apply an *observational causal inference* approach based on propensity score matching^23^. The main idea consists of identifying pairs of situations that are *similar* but for the self-disclosed gender of their authors. This matching ensures that we fairly compare judgments of similar submissions, thus getting closer to the estimate provided by the ideal experimental setting.

This setting requires to first identify the causal mechanism that makes the self-disclosed gender affect the community judgment, which we summarize in the causal graph in Fig. 1. Our first assumption is that the community deliberate based only on information contained in the text of the submission. We believe this assumption is realistic given the functioning of /r/AITA. More concretely, this assumption means that the community makes judgments only based on the text of submissions, which contain the self-disclosed gender and age of the author and present a first hand experience of the author. This is reflected in Fig. 1 from the arrows that point to “*Judgment*”. Indeed, different situations may have their own likelihood per-se to be judged to deserve blame (e.g., not leaving the seat to an old person in public transport being very likely to be judged negatively; see the path between “*Experiencing a situation*” and “*Judgment*” in Fig. 1). In addition, gender and age can affect the spectrum of situations someone can experience (e.g., pregnancy-related situations experienced by women, or work-related situations less likely to be experienced by teenagers; see the links from “*Gender*” and “*Age*” to “*Judgment*” in Fig. 1) as well as the propensity to share an experience on social media (see the links “*Gender*” and “*Age*” to “*Posting on* /r/AITA” in Fig. 1). Finally, prior work shows that age can interact with gender to shape moral judgments^7,29^, which motivates the use of age as a control variable.

Our second assumption states that all submissions are judged by a homogeneous jury, that is, the set of judges on any given story is a random sample from the population of judges. This assumption reflects an intrinsic limitation of Reddit, as the platform does not provide access to the identities of the users who upvote comments, thus making it impossible to identify the judges.

Building on these assumptions and our goal of controlling for both the situation and the author’s age, we employ *propensity score matching* to compare similar pairs of male- and female-authored submissions. In the following subsections, we describe how we train the propensity scorer, how we implement the matching, and how we evaluate these matched pairs.

### Training the propensity scorer

The propensity scorer predicts the probability that the author is male. Then, this probability is used to match pairs of submissions whose propensity score is similar, indicating that the pair is similar in terms of the likelihood that the situation can happen to a male or a female. We employ a BERT model^30^ as the propensity scorer since transformer-based models have been found to outperform other text representations in causal studies^31^. We fine-tune BERT using the concatenation of the title and text of the submission as input and the self-disclosed gender of the author as target. Since the gender of the protagonist is extracted from the demographic tags present in the text, we remove all the demographic tags from the text.

To mitigate the model’s reliance on gendered words as shortcuts for predicting authors’ gender (e.g., the expression “my wife” being highly predictive of the protagonist being male), we implement a “gender neutralization” strategy^32^. This strategy consists of swapping all gendered words in a submission with a 50% probability during training (e.g., “wife” changed to “husband”; see Supplemental Material for additional details). This approach is crucial because features that predict exposure (i.e., declared gender in our case) can be problematic in causal inference^23^.

We train the model (*bert-base-uncased*) for three epochs using a learning rate of $2 \times 10^{-5}$, batch size 64, weight decay 0.01, linear warm-up for $10\%$ of training steps, and class weighting reflecting the relative fraction of male- and female-authored submissions in the training set. We employ an early stopping strategy on $10\%$ of the training set. Such parameters were chosen after a 5-fold cross-validation on the learning rate ($2 \times 10^{-5}$, $5 \times 10^{-5}$) and the number of epochs (3, 5), which resulted in negligible differences.

### Matching

To match pairs of similar submission, we introduce a definition of distance that combines the propensity score and the age difference between the authors. Specifically, the distance between two submissions is defined as the semantic distance between the two, provided that they have a similar propensity score and the age difference of the authors is small. We use a document embedding model, SBERT^33^, to encode submissions into a semantically meaningful vector and compute document pairwise distance through cosine distance. The employment of this definition of distance in the matching allow us to pair submissions that are similar in terms of both semantic content (i.e., the situations described) and propensity score.

More formally, the distance $D_{i,j}$ between a pair of submissions is defined as:

$$\begin{aligned} D_{i, j} = {\left\{ \begin{array}{ll} 1 - \cos \left( \vec {i}, \vec {j} \right) , & \text {if} \; \left| e\left( i \right) - e\left( j \right) \right| < c \\ & \wedge \; 1 - \cos \left( \vec {i}, \vec {j} \right) \le D_{\max } \\ & \wedge \; \tau _i = \tau _j \\ & \wedge \; \left| age\left( i\right) - age\left( j\right) \right| \le \delta \\ & \\ + \infty , & \text {otherwise} \end{array}\right. } \end{aligned}$$

where $\cos \left( \vec {i}, \vec {j}\right)$ is the cosine similarity between the submission embeddings of submissions *i* and *j* obtained via SBERT, $e\left( i\right)$ is the logit of the propensity score of submission *i*, $\tau _i$ is the topic of submission *i*, *c* is the caliper, $D_{\max }$ is the maximum matching distance, $age\left( i\right)$ is the age of the author of submission *i*, and $\delta =5$ is the maximum difference in age between authors of submissions. The caliper is determined as $c = 0.2 \times \sqrt{\frac{\sigma ^2_T + \sigma ^2_U}{N_T + N_U - 2}}$, where $\sigma ^2_T$ ($\sigma ^2_U$) is the variance of the distribution of the logit of the propensity score of submissions authored by males and females, respectively^34^.

Once all pairwise distances between pairs of submissions are computed, we obtain 1:1 matches without replacement via the minimum weight matching algorithm, which minimizes the sum of the distances between the matched submissions. We opt for 1:1 instead of 1:many matching because it simplifies the manual evaluation, as we describe in the Section “Evaluating the quality of matched submissions”. We consider different values of $D_{\max }$ from 0.15 to 0.35.

We estimate the effect of the gender on the received judgment through the sample average treatment effect on the treated (SATT)^35,36^, defined as:

$$\begin{aligned} SATT = \frac{1}{N_T} \sum _{i=1}^{N_T} Y_i\left( M\right) - Y_i\left( F\right) \end{aligned}$$

where $N_T$ represents the number of submissions written by male authors with a match, $Y_i\left( M\right)$ is the community judgment of the *i*-th male-authored submission (1 if judged deviant, 0 otherwise), and $Y_i\left( F\right)$ is the outcome of the female-authored submission (i.e., written by a female author) that has been matched to the *i*-th male-authored submission. In other words, the SATT quantifies how the community judges male authors when they share narratives akin to those shared by female authors. Unlike the population average treatment effect, SATT estimates the effect on a pruned sample of submissions for which we can find a match satisfying the semantic constraints, and should not be used to infer the effect on the larger population (i.e., the whole subreddit)^35,37,38^. In other words, SATT estimates the effect in the population of submissions for which male and female protagonists share comparable narratives, in line with the purpose of this study.

### Evaluating the quality of matched submissions.

Matching methods typically check for covariate balance to assess the quality of the matched sample^23^, but encoding textual data into high-dimensional spaces may invalidate these diagnostics^39^. Nonetheless, unlike other types of data, texts can be easily interpreted by humans, thus allowing for the manual assessment of the quality of the matches by conceptualizing a notion of similarity^35,39^, albeit introducing some subjectivity in the task.

We thus employ a manual annotation process to evaluate the quality of the matched submissions. The annotation consists of three steps for each pair of submissions. In the first and third steps, the annotator is asked to assess the level of agency of the two protagonists. After reading the title and body of each submission, the annotator is asked to reply to the question “*How much of the whole event in the text is caused/initiated by the author?*”^40^ on a 5-point Likert scale ranging from *Not caused by the author* to *All caused by the author*. A low level of agency refers to authors who only react to others—for instance, being unfairly blamed—whereas a high level of agency is given when the situation would not have occurred in the same way without the action of the author, such as saying something that directly triggers a conflict. The interest in this information is related to the second hypothesis about the observed association between gender and judgment. That is, if male protagonists share more morally ambiguous situations than female ones, we should expect male protagonists to express a higher level of agency. In the second step, the annotator is asked to evaluate the similarity between the two stories on a 5-point Likert scale ranging from *Very dissimilar* to *Very similar*.

Five raters familiar with the /r/AITA dataset annotate a random sample of matches, with each match annotated by 3 different annotators. Despite their familiarity with the dataset, annotating for agency and the pairwise similarity of stories is a difficult task. Indeed, submissions can describe stories that are convoluted and rich in details, with overlaps on some narrative elements while diverging on others. We therefore ran a preliminary training phase where each rater first annotated a small set of pairs, after which they met to compare judgments and agree on shared guidelines. During this discussion, the raters clarified, for instance, that hypothetical “What if I did ..” posts—where the author has not yet acted—should be scored in terms of agency as if the action had occurred, ensuring consistency with cases where the author actually acted. Annotators also refined a common understanding of similarity between stories that balances multiple aspects such as the persons involved (e.g., family, friends, colleagues), the specific actions, the roles of each participant, and the setting when relevant. Thus, a story in which the protagonist yells at a family member is considered dissimilar from one where the family member yells at the protagonist: despite the individuals involved and the action being the same, the roles are reversed. As another example, consider a pair of stories consisting of one where the protagonist yells at a friend at home, while the other where the protagonist yells at a friend in a public place, such as a restaurant. Here, the similarity of characters and actions is outweighed by the difference in places where the action unfolds, so the stories are considered dissimilar.

After the annotation step, we employ median aggregation to the ratings on the similarity between the pairs. That is, given the annotations from the three raters to each pair of submissions, we discard the two most extreme judgments. In cases where the aggregation resulted in “Neither dissimilar nor similar”, the authors of this study engaged in conflict resolution.

## Results

The data collection results in a total of 33421 submissions annotated for judgment and demographics: 21.612 authored by females ($65\%$), while 7269 judged to deserve blame ($22\%$). Nearly half of the submissions are authored by users aged 19 to 26 for both male and female authors (see Supplementary Fig. S3).

### Male authors are more likely to be judged deviant

In line with previous studies, we start by measuring the association between gender and judgment. Our crude estimate aligns with these studies: male authors are approximately twice as likely as female ones to receive a negative judgment from the community (OR $= 2.21$, $95\%$ CI: 2.10–2.33, Fisher’s exact test $p\text {-value} < 0.001$). This association is consistent across different topics and age groups (see Supplementary Figures S5 and S6, respectively). Thus, we confirm that male authors receive negative judgments more frequently compared to female authors, regardless of context and age. However, according to our second hypothesis, this correlation might be the result of male and female authors being different in terms of their propensity to share morally ambiguous situations. In the following, we address the problem of measuring the causal effect of the gender on the received judgment.

#### Situations explain away the difference

**Fig. 2:**
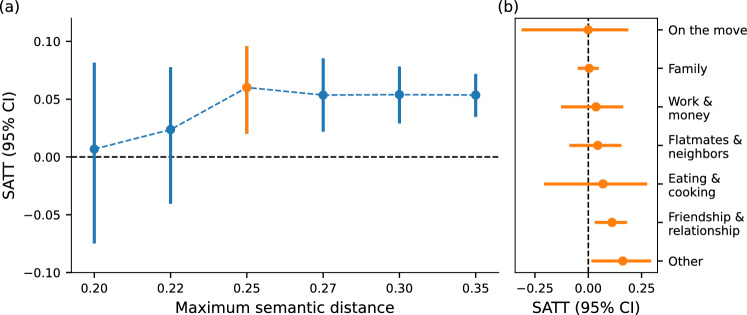
Causal effect of the protagonist’s gender on moral judgment. (**a**) Sample average treatment effect on the treated (SATT) for different values of the maximum semantic distance. We report the SATT from maximum semantic distance equal to 0.20 because of the small amount of matches below this value ($N<120$). (**b**) SATT for each topic corresponding to the matches obtained with maximum semantic distance equal to 0.25. Vertical bars correspond to bootstrap 95% confidence intervals.

Our analysis reveals weak to no evidence supporting a gender bias in community judgments when the narratives are similar. Figure 2a shows how the SATT varies for different values of the maximum semantic distance. The effect of gender is small and not statistically significant for small values of the maximum semantic distance. However, as the maximum semantic distance increases, a small yet significant effect emerges (SATT $= 0.06$, $95\%$ CI: 0.02–0.10) in a matched sample composed of 699 pairs of submissions ($6\%$ of submissions authored by male protagonists). For larger maximum matching distances, the SATT does not increase further.

To contextualize the SATT obtained at a maximum semantic distance of 0.25, we repeat the initial association test on the subset of matched submissions. Indeed, matching can be used as a preprocessing approach to clean the data by selecting a subset of items such that treatment and control units are similar^41^. By doing so, we can assess how the association differs from the one measured in the whole dataset. In the matched sample, male protagonists are 1.52 times more likely to receive a negative judgment ($95\%$ CI: 1.15–2.01, Fisher’s exact test $p\text {-value} = 0.003$), compared to 2.21 in the whole dataset. This change indicates that our matching approach has identified a subset of submissions where the association between gender and judgment is 1.45 times smaller than in the whole dataset, thanks to the selection of narratives shared by both male and female protagonists. The difference between the two odds ratios is deemed significant according to the Breslow-Day test ($\chi ^ 2 = 7.73$, *p*-value $= 0.005$).

Next, we stratify the SATT across the topics identified by LDA to understand if there is a differential contribution of the topic to the observed SATT. Figure 2b shows the values obtained for maximum matching distance equal to 0.25 (the behavior of each topics’ SATT as a function of the maximum semantic distance is similar to the one in Fig. 2a, as can be seen in Supplementary Fig. S7). For most of the topics, the SATT consistently hovers around zero. The small sample sizes may hinder the ability to measure small causal effects. For instance, topics such as “Eating and cooking” and “On the move” comprise 29 and 16 matched pairs, respectively, for a maximum semantic distance of 0.25. In contrast, topics like “Friendship and relationship”, “Family”, and “Other” (i.e. the miscellaneous category) have a larger number of matches. Two of them (“Friendship and relationship” and “Other”) align with the overall pattern of the SATT depicted in Fig. 2, where the SATT is not significantly different from zero for small maximum semantic distances but becomes positive for thresholds of 0.25 or larger. However, the SATT of the “Family” topic is not significantly different from zero despite being the largest topic, suggesting that the small observed gender bias may manifest differently depending on the context. Overall, the SATT measured on the whole matched sample is mainly driven by one single topic, “Friendship and relationship”.

#### The exceeding bias is due to more distant matches

Our findings illustrate a weak or absent gender bias in moral judgment and rely on textual embeddings derived from Large Language Models to identify pairs of similar submissions. However, the quality of such pairs may confound our findings. Indeed, pairs of submissions that do not represent similar situations might carry the crude *male-blame* association observed in the whole dataset, thereby escaping our efforts to control for biases.

To evaluate the quality of matched pairs, we conduct a manual evaluation of the matches obtained with a maximum matching distance of 0.25. This threshold represents the most conservative value that produces a significant gender bias in judgments. A random sample of 100 matches was annotated (16% of the matched submissions). The inter-annotator agreement, measured by Krippendorff’s alpha ($\alpha =0.42$), indicates agreement to some extent among annotators despite the subjective nature of the task. After aggregating the scores, 63% of matched pairs received a score of 4 or higher (i.e., “*Somewhat similar*” or “*Very similar*”), while 28% scored 2 or lower (i.e., “*Somewhat dissimilar*” or “*Very dissimilar*”), with the remainder scoring 3 (i.e., “*Neither dissimilar nor similar*”). The result of the manual evaluation indicates that the situations described in the matched pairs of submissions are similar in most of the cases. We provide examples of matches submissions for different levels of similarity as assessed by the annotators in Table S4.

**Fig. 3:**
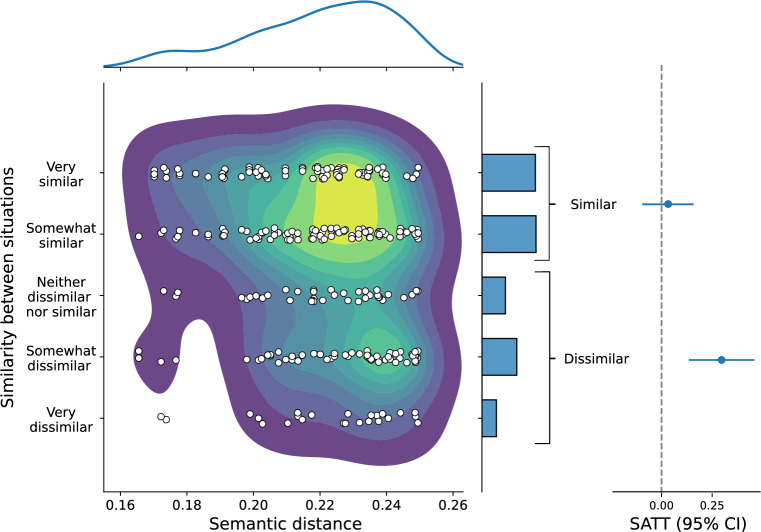
Joint distribution of annotator judgments and distance between matched submissions obtained with maximum matching distance at 0.25. Dots correspond to single annotations of matched submissions (not the aggregated judgment through median aggregation) and the joint plot is obtained through kernel density estimation. Colors range from purple (low density) to yellow (high density). The marginal distributions are shown on top and on the right. On the right, SATT and 95% confidence interval obtained separately for the matched pairs evaluated as similar or dissimilar.

To further understand if the matching procedure effectively reflects the concept of situational similarity, we measure the correlation between the semantic distance and the scores given by annotators. Figure 3 shows the joint distribution of annotators’ judgments (disaggregated) and the semantic distance between the matched submissions. For small distances, the evaluations concentrate around “*Very similar*” and “*Somewhat similar*” (i.e., 5 and 4 on the Likert scale), indicating that the similarity between situations is effectively captured by a small semantic distance between the submissions. Instead, dissimilar pairs start to be matched for semantic distance larger than 0.20. A correlation test between semantic distance and human evaluations finds no evidence for such a relationship (Kendall Tau-b $= -0.10$, $p\text {-value} = 0.17$), suggesting no significant relationship between the similarity of situations and the semantic distance. However, the matching procedure is still able to identify effectively similar situations.

Despite the majority of pairs representing similar situations, the presence of a 37% of dissimilar ones may distort the SATT towards positive values. To test this intuition, we estimate the SATT for the similar and dissimilar pairs of submissions separately. As expected, we observe that similar matches produce a SATT that is not statistically different from zero (SATT=0.03, 95% C.I. $-0.10$–0.16), whereas dissimilar ones result in a positive effect (SATT=0.30, 95% C.I. 0.14–0.46), as shown in Fig. 3. Therefore, the positive and significant gender bias observed for the semantic distance threshold at 0.25 or higher is likely a residual effect due to the presence of dissimilar matched pairs, which contribute positively to the SATT. Thus, these observations point to a lack of gender bias in moral judgments on /r/AITA.

## Discussion

In this study, we measure the causal relationship between the self-disclosed gender of users sharing morally ambiguous situations and the moral judgment received from the Reddit community. Our study design leverages moral judgments from /r/AITA, which has two main advantages. First, Reddit users are likely to share sensitive stories and honest judgments as their accounts are pseudonymous^42–44^. Second, the voting mechanism of /r/AITA taps into the wisdom of the crowd and allows a large number of users to express their opinions on the specific situation depicted in the submission. This, in turn, provides the analysis with a large sample of judgments. We have found that most of the apparent effect that makes male protagonists more likely to receive a negative judgment^10,11,13^ disappears when controlling for the situation described in the post. That is, for situations shared by both males and females, disclosing the gender of the author does not directly affect the judgments received. This result mirrors other work, which likewise observed no gender penalty when only the agent’s name was varied in classic trolley dilemmas^5^—even though those dilemmas are highly stylised and far from the real-world conflicts posted on /r/AITA. This effect survives only in the subset of submissions related to friendship and relationships.

One potential explanation for our result is that males tend to describe ‘riskier’ situations than females. Indeed, the financial and economic literature has repeatedly observed that “women are more risk-averse than men”^18^. Similar results have been found by sociologists and psychologists^16,45^. If we assimilate disclosing a personal story that might violate social norms and cause discomfort with risk-taking, this evidence suggests that women might self-censor more often than men when they believe they have crossed the line. Indeed, there is evidence that men are more *overconfident* in their behavior, and this overconfidence translates into a lower return on their investments^17,46^. Although we did not measure this aspect, a similar phenomenon might be at play in our case: men are overconfident in their understanding of social norms. This overconfidence may lead them firstly to put themselves in ‘dubious’ situations and secondly to be more likely to share them, thus receiving an overall more negative judgment. Under this interpretation, the disparity arises from what gets posted rather than from bias on the part of the judges.

A complementary explanation stems from differences in how women and men use online fora. Broadly speaking, and conscious of the risk of stereotyping, men tend to use online groups primarily for information seeking, while women for encouragement and support while sharing their personal experiences^19^. Indeed, women offer more social support on online social networks^20^, and engage more often with coping strategies when dealing with distress, e.g., through verbal expression of emotions to seek social support^47^. Women also more easily provide social support, whereas men (who emphasize achievement, autonomy, and emotional control) have a harder time in seeking and obtaining social support^21^. These differences might explain both why males are overconfident and how women use the community as a social support group^13^. Indeed, the community is aware of the tendency to use the subreddit for *validation*, and has been actively discussing it^48^.

Our findings and the supporting literature should also be considered in light of rise of AI assistants based on LLMs^49–51^. These models are trained on publicly available user-generated data such as Reddit. As with every algorithmic automation, a chatbot assistant trained on biased data would amplify and reinforce existing behaviors unfair towards specific subpopulations. Clearly, this can be harmful per se. Still, the scenario might further spiral down as more automatically generated text (trained on biased data) will be used to train novel LLMs with even stronger biases. Thus, our study highlights additional potential risks in deploying and using LLMs to augment, or even automate mental health support services^52^. At the same time, using a causal approach in the machine-learned model might be a viable strategy to remove the apparent bias.

We have found that disclosing the gender of the author does not affect the moral judgment received, so where does the observed *male-blame* association come from? Based on our causal assumptions depicted in Fig. 1, the other causal path from the author’s gender to judgment passes through the likelihood of sharing a specific situation based on the authors’ gender. A hint for this mechanism being in place comes from secondary findings derived from the manual annotation, where we observe that male authors are more agentic than female ones among the pairs of submissions judged dissimilar (see Supplementary Table S2 and Supplementary Section “Additional results of the manual evaluation”). Agency is indeed a primary antecedent of negative moral judgment^15^ and it is stereotypically associated with males^53^. If agency both covaries with gender, as our results suggest, and amplifies blame, then the “male-blame” association we observe could be at least partly attributed to agentic language rather than gender per se. In other words, this observation points to male protagonists being more responsible for the event than female ones, and possibly for a higher likelihood of males sharing morally ambiguous situations, if we assume that receiving negative judgments correlates with higher agency (see Supplementary Table S3). Testing this mechanism is a promising direction for future work, which requires quantifying the level of agency of protagonists at scale.

Our findings should be interpreted in light of three main limitations. First, our work is based on the causal graph depicted in Fig. 1. Although we designed the causal graph by reviewing potential mechanisms discussed in relevant works, we cannot exclude the presence of other mechanisms that mediate the effect of gender on judgment. For instance, potential homophily effect whereby judges favour authors of their own gender. Although possible in principle, measuring this effect is not possible given the nature of the data because Reddit does not provide information on users who upvote, a fundamental mechanism to express users’ judgment. A second limitation stems from our choice to consider only submissions where a demographic tag is present. This provides us with a reliable, explicit indication that can be extracted with a high level of accuracy. However, we excluded other posts where judges may infer authors’ gender implicitly. If such posts differ systematically from the ones containing the demographic tag in how judges make judgments, our estimate could be biased. In addition, our study does not account for potential interaction effects with the gender of the involved persons, which can mediate the relationship between authors’ gender and judgment. Finally, determining whether two stories describe similar situations is unavoidably a subjective task. Indeed, the described stories are often complex and rich in details, making it challenging to identify the aspects that matter the most to assess the similarity between pairs of stories. This challenge is reflected in the moderate agreement between annotators. We therefore based our conclusions on the subset of matched pairs for which the annotators agreed on the similarity of the submissions.

While many gender biases remain^54–56^, our result contributes to a body of hopeful results that show their absence^57^. We may speculate that a generational change affects these biases, as “Gen Z”, which composes the primary user base of Reddit, is known to be more sensitive to gender issues^58^. Overall, our results have important implications for gender studies, the study of social norms, and the understanding of socio-technical systems.

### Ethics statement

We declare that it is an original work, and it has not been published elsewhere nor is under consideration by another journal or conference. All authors have approved the manuscript and agree with its submission. The authors comply with the research ethics guidelines of ACM Code of Ethics and Professional Conduct for computer sciences. This research involved the retrospective analysis of publicly available data from Reddit and was therefore exempt from approval by the Institutional Review Boards at the authors’ institutions. The users and moderators whose contributions were analyzed were fully aware of the public nature and free accessibility of the content they posted, as the subreddits examined are publicly accessible, not password-protected, and have thousands of active subscribers. Moreover, Reddit’s pseudonymous accounts make the retrieval of users’ true identities unlikely. Therefore, our research did not require informed consent. To maximize privacy and minimize potential harm, all results were reported in aggregate.

## Supplementary Information

Supplementary Information.

## Data Availability

The datasets generated and the scripts used for the analysis are available in the Zenodo repository, https://zenodo.org/records/13305890.
