# Supplementary material for: Moral judgments in online discourse are not biased by gender
Source: Sci Rep. 2025 Jul 1;15:21555. doi: 10.1038/s41598-025-08749-x (PMC12219380; doi:10.1038/s41598-025-08749-x)
Supplement: Supplementary file 1 — Supplementary Information. [file 41598_2025_8749_MOESM1_ESM.pdf]

Supplementary Material of: Moral Judgments in Online Discourse are not Biased by Gender

Lorenzo Betti[1,2,*], Paolo Bajardi[3], and Gianmarco De Francisci Morales[3]

[1]ISI Foundation, Turin, 10126, Italy
[2]Central European University, Department of Network and Data Science, Vienna, 1100, Austria
[3]CENTAI, Turin, 10138, Italy
[*]lrn.betti@gmail.com

Bot detection

We discard content published by bot accounts, including two bots used by `/r/AITA` moderators to perform automatic tasks: `AutoModerator` and `Judgement_Bot_AITA`. We refer to the subreddit `r/BotDefense`, where users report other users exhibiting suspicious behavior and the subreddit assigns a flag to the reported user if it meets certain criteria. We collect all the submissions in this subreddit and classify as bots all the users for which the service assigned the flair "banned", "declined", or "service". This amounts to 2604, 429, and 98 users respectively. We discard all the contents produced by this set of users (i.e., both submissions and comments).

Data collection

We collect all the submissions and comments on `/r/AITA` from the beginning of 2014 to the end of 2020 from the Pushshift Reddit data collection. We filter out all submissions whose title does not start with "AITA" (i.e., "Am I the asshole") or "WIBTA" (i.e., "Would I be the asshole"), since authors who want to receive community judgment must start the tile with such tags according to community guidelines. In addition, we discard submissions whose final judgment is "NFO", indicating that the author did not provide enough information to let the community deliberate. Submissions whose body was deleted or removed are discarded as well. Then, we collect the comments under the filtered submissions containing at least one judgment tag. After the removal of contents published by bot accounts, the dataset contains 252 269 submissions and 8 191 812 comments.

In the next step, we extract the judgment tags from the comments. Users do not always use judgment tags only to express their judgment. For example, they can discuss the behavior of the author and use judgment tags as abbreviations, instead of using them with intent of expressing their judgment. In addition, the "NAH" tag may be confused with the informal spelling of "no", since users use tags with various case variants (e.g., "NAH" as well as "Nah" and "nah"). To extract judgment tags from comments whose intent is to cast a judgment on the behavior of the author, we develop a set of rules by considering where the tag occurs within the comment:

1. The tag is the only word in a line, irrespective of the case. In this case, the tag is separated from the comment body and we can safely assume that the judge intends to use it to express their vote. This is the only case in which we considered the tag even in the presence of other tags in the comment.

2. The tag is the only word of a sentence, irrespective of the case. Similarly to the previous rule, the author chooses to isolate the tag from the body of the comment. In this case, we do not consider the tag "Nah" and "nah" because of the ambiguity with the informal spelling of the word "no". However, this happens only in approximately 8k comments.

3. The tag is the first word of a line and written in upper case, or it is followed by some special characters irrespective of the case. In this scenario, the upper case or the presence of a special character isolates the tag from the rest of the comment, similar to the previous cases. The special characters we consider are: ".", "-", "(space)-", ";", ":", "(space):", "(double space)".

4. The tag is written in upper case in a sentence composed of at most 6 words. This rule covers a non-negligible fraction of cases in which the judge wants to emphasize their judgment. For example, "OP, you are clearly NTA!", "Uh yeah, YTA", and "I think ESH". As an exception to this rule, we discard all cases where the short sentence contains the word "if" or ends with a question mark, as in these two cases the sentence may be hypothetical or a question.

Each rule is responsible for selecting 17%, 31%, 32%, and 5% of comments, respectively, while the remaining 15% of comments are discarded. Approximately half of the discarded comments are removed because they have a negative score (difference between upvotes and downvotes). We decided to remove them as they are likely to violate the rules of the community. Finally, after having removed submissions with no evaluations, our dataset comprises 250 770 submissions and 6 891 476 judgments.

Topic detection

Table S1. Top 10 words for the identified topics.

Topic	Top 10 words
Eating and cooking	eat, food, wear, buy, thing, dinner, hair, drink, cook, feel
On the move	play, car, game, drive, walk, start, minute, leave, phone, watch
Flatmates and neighbors	dog, room, house, live, home, leave, sleep, day, clean, roommate
Friendship and relationship	friend, feel, talk, thing, good, people, start, year, day, girl
Family	mom, family, dad, sister, year, parent, brother, kid, mother, husband
Work and money	work, pay, money, job, day, week, year, month, buy, school

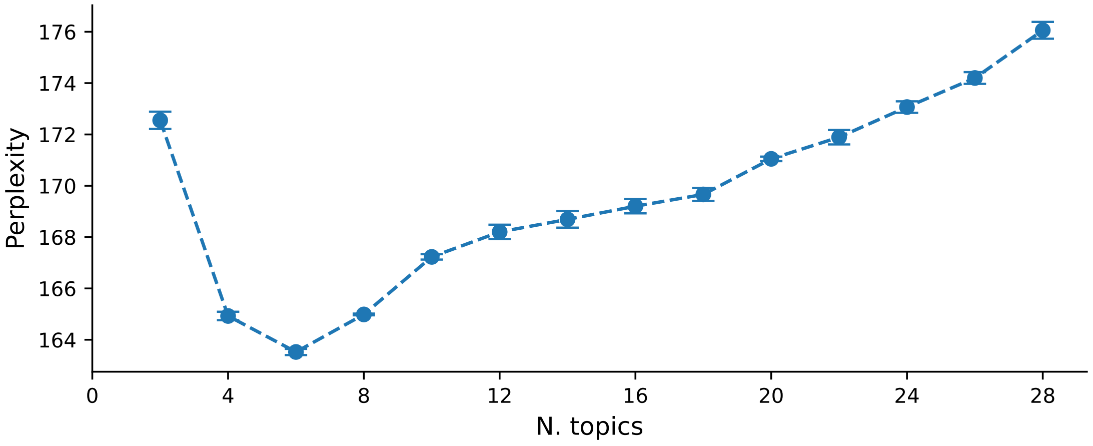

Figure S1. Perplexity on held-out set. Circles indicate the mean and vertical bars indicate the standard error of the mean of the perplexity obtained in a 5-fold cross-validation.

Extraction of author demographics

We extract the demographic information of the authors from their submissions. Authors often use demographic tags to provide more context to their stories. We develop a regular expression to extract tags that contain information about the author's age and gender. The regular expression selects demographic tags in the proximity of first-person singular pronouns (e.g., "I am a F26 [...]", "My (F26) [...]") and handles variations of the patterns such that the gender tag can be before or after the age, and possibly separated by a whitespace (e.g., "F 26", "26 F", or "26F"). In total, 39 005 submissions contain this demographic tag, accounting for 15.6% of the submissions.

To account for other gender identities, we modify the regular expression to find strings containing a two-digit number (representing the age) followed or preceded by up to four capital letters, which might potentially refer to other gender tags. We manually inspect tags occurring more than 2 times, and find acronyms related to non-binary genders (NB) and transgender (MTF or FTM, meaning users transitioning from male to female or female to male respectively), just to name a few. After including also case-insensitive versions (e.g., MtF) and variations (e.g., M2F), we identified 309 additional submissions with other gender tags. Since these constitute less than 1% of the total users with available demographic information, we consider only the binary categorization of gender (M and F) due to the limited data on other gender identities.

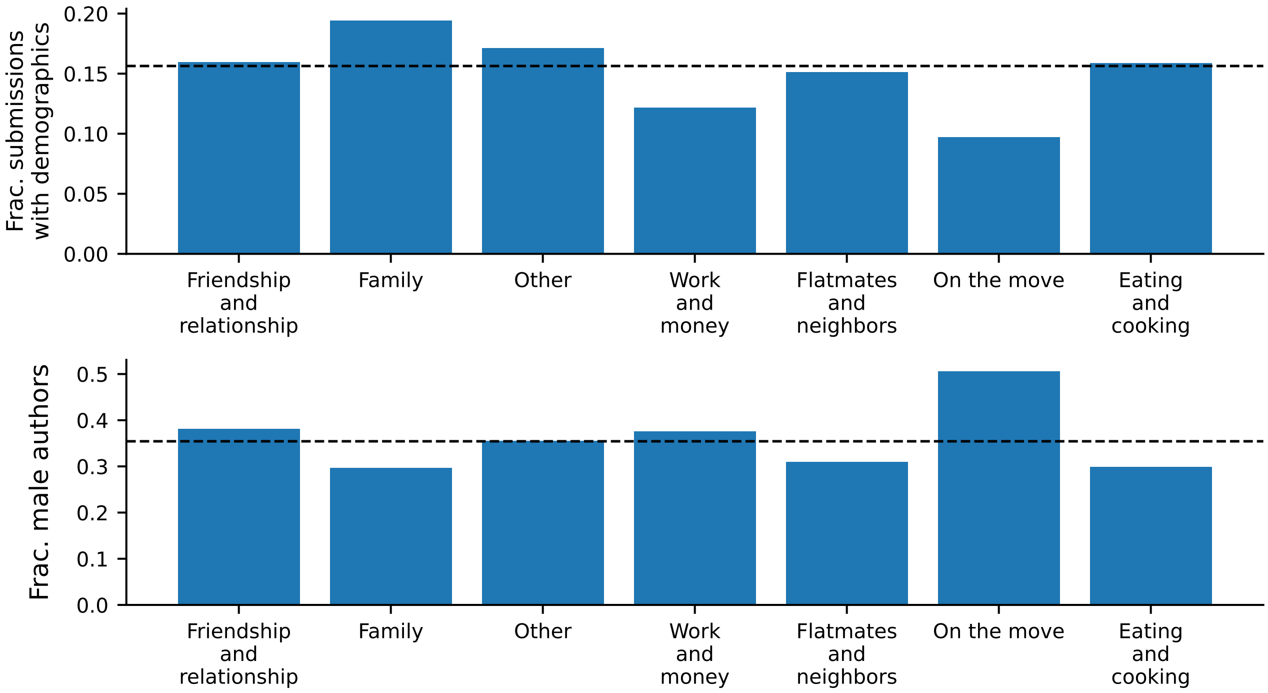

Figure S2. Distribution of submissions with available demographics (top) and male authors (bottom) for each topic identified by LDA. The black dashed line refers to the fraction of submission from which it has been possible to extract the demographic tag (top) and the fraction of male authors (bottom).

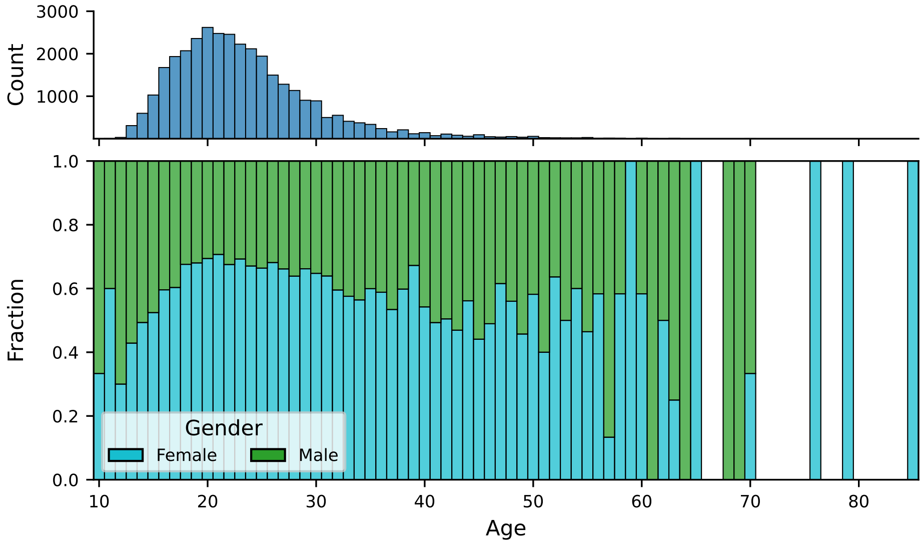

Figure S3. Fraction of male and female authors for different ages. The top plot shows the aggregate distribution of authors' age.

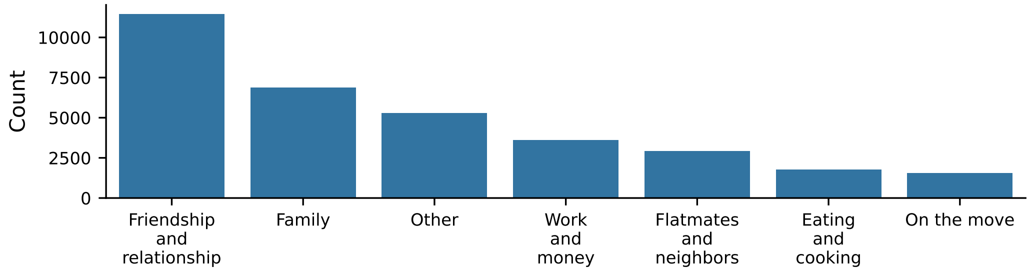

Figure S4. Distribution of topics in the AITA dataset as returned by the LDA topic model. Submissions are assigned to the topic with highest probability if higher than $t_{topic} = 0.40$. Otherwise, they are assigned to the "Other" topic.

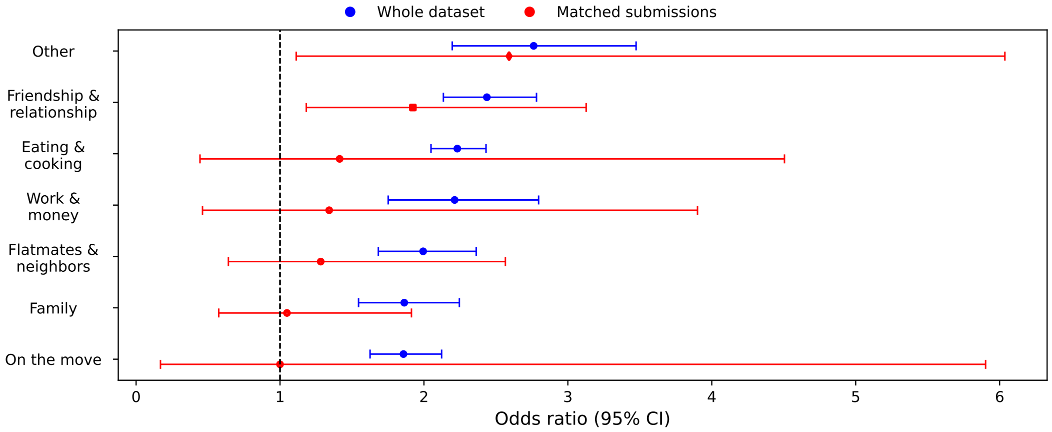

Figure S5. Odds ratio of receiving a negative judgment given the gender of the author for each topic separately. Results show separately the associations for the whole dataset (blue lines) and the matched sample (red lines). Values larger than one indicate that male authors are more likely to receive negative judgments. Horizontal bars refer to 95% confidence intervals. Empty markers indicate lack of significance at 0.05 level.
$\diamondsuit : p < 0.05$, $\square : p < 0.01$, $\bigcirc : p < 0.001$

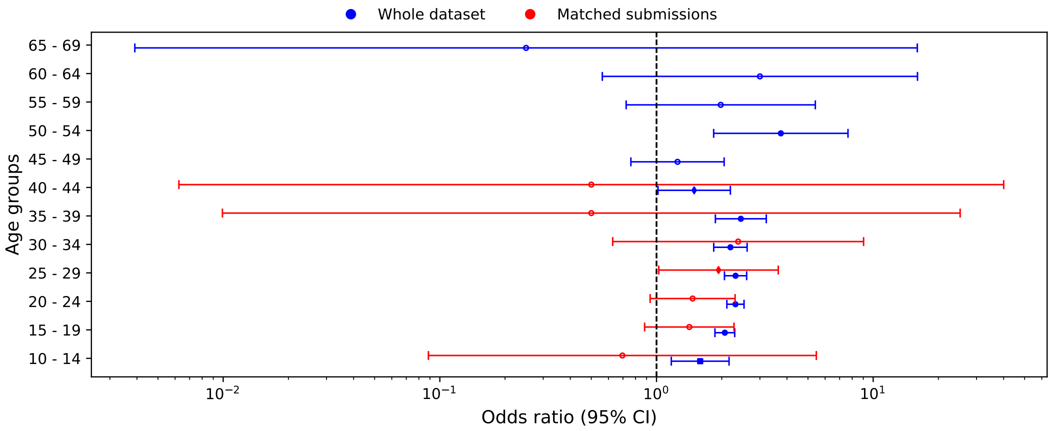

Figure S6. Odds ratio of receiving a negative judgment given the gender of the author for different age groups. Values larger than one indicate that male authors are more likely to receive negative judgments. Horizontal bars refer to 95% confidence intervals. Empty markers indicate lack of significance at 0.05 level.
$\Diamond$: $p < 0.05$, $\Box$: $p < 0.01$, $\bigcirc$: $p < 0.001$

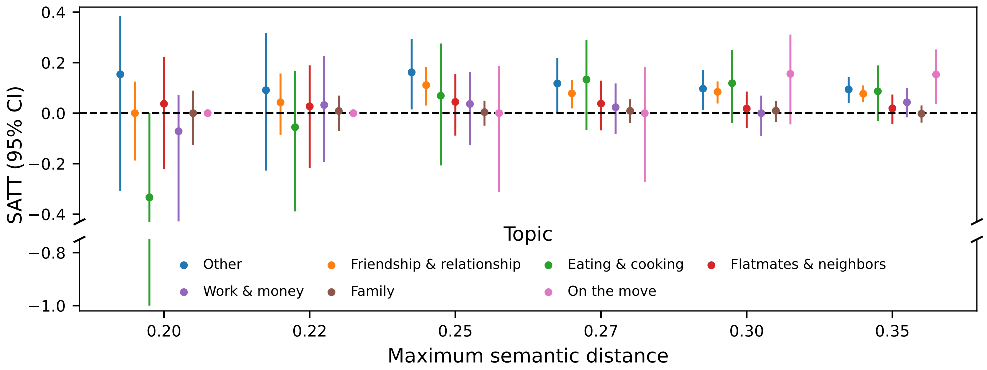

Figure S7. Average treatment effect on the treated (ATT) as a function of the maximum matching distance for each topic separately. Vertical bars correspond to bootstrap 95% confidence intervals.

Additional results of the manual evaluation

The main purpose of the manual evaluation is to validate the matching procedure that identifies submissions describing similar situations. In addition to this, we ask the annotators to evaluate the level of agency of the authors of the submissions, as described in the Methods section. The analysis of the authors' agency in relation to their gender and moral judgment provides validation to the manual annotation and supports the discussion of our findings.

First, we test to what extent the level of agency is related to the self-disclosed gender of the author and the moral judgment received. We fit two linear mixed-effects models:

$$\text{Initiator\_score} \sim \text{Gender} * \text{isDissimilar} + (1 \mid \text{Annotator})$$
$$\text{Initiator\_score} \sim \text{Judgment} * \text{isDissimilar} + (1 \mid \text{Annotator})$$

where isDissimilar is a dummy variable that has value 1 if the submission belongs to a match evaluated as dissimilar, the term $(1|\text{Annotator})$ refers to a random intercept associated to each annotator, and the "$*$" indicates that the model considers both variables and their interaction. The isDissimilar variable indicates whether the submission belongs to the group of submissions that have a match considered to be similar. The coefficient corresponding to the interaction term estimates the differential effect of the predictor (i.e., gender and judgment) in the subset of submissions that result from a wrong match.

We present the result of the first model in Table S2. Although the model returns no significant overall contribution of the gender ("Gender"), the interaction term between gender and isDissimilar has a positive and statistically significant coefficient ("Gender:isDissimilar"), meaning that male authors have a higher initiator score when the submission belongs to a match judged to be dissimilar. In plain terms, there is no significant difference in the level of agency of male and female authors for submissions judged to be similar, whereas male authors have a higher level of agency for dissimilar submissions. This result suggests that the level of agency of the protagonist is effectively controlled in the subset of correctly matched submissions, whereas male protagonists are more agentic than female ones in the subset of submissions with a dissimilar match.

Table S2. OLS regression model of author's gender on initiator score.

Model:	MixedLM	Dependent Variable:	Initiator_score
No. Observations:	600	Method:	REML
No. Groups:	5	Scale:	1.1004
Min. group size:	120	Log-Likelihood:	-889.0219
Max. group size:	120	Converged:	Yes
Mean group size:	120.0		

	Coef.	Std.Err.	z	P> |z|	[0.025	0.975]
Intercept	1.567	0.159	9.884	0.000	1.256	1.877
Gender	0.095	0.108	0.883	0.377	-0.116	0.307
isDissimilar	-0.262	0.126	-2.078	0.038	-0.508	-0.015
Gender:isDissimilar	0.427	0.177	2.409	0.016	0.080	0.775
Group Var	0.096	0.071				

Table S3 reports the result of the second model. Here, negative moral judgment is associated to a higher level of agency ("Judgment"), while there is no significant differential effect with respect to the fact that the submission belongs to a match judged dissimilar ("Judgment:isDissimilar"). This result points to higher levels of agency associated to a higher likelihood of receiving a negative moral judgment.

Table S3. OLS regression model of moral judgment on initiator score.

Model:	MixedLM	Dependent Variable:	Initiator_score
No. Observations:	600	Method:	REML
No. Groups:	5	Scale:	1.0612
Min. group size:	120	Log-Likelihood:	-877.7939
Max. group size:	120	Converged:	Yes
Mean group size:	120.0		

	Coef.	Std.Err.	z	P> |z|	[0.025	0.975]
Intercept	1.482	0.152	9.741	0.000	1.184	1.781
Judgment	0.692	0.135	5.128	0.000	0.428	0.957
isDissimilar	-0.019	0.097	-0.201	0.841	-0.209	0.170
Judgment:isDissimilar	-0.101	0.227	-0.444	0.657	-0.545	0.344
Group Var	0.098	0.074				

Table S4. Examples of matched submissions. The third column reports the evaluations given by the annotators.

Submission 1	Submission 2	Evaluations
Today, my mom [38F] asked me [17M] for 50PLN. It's a lot for me since I have limited pocket money. She's unemployed and looking for a part-time office job. She avoids telling me why she needs money, often for cigarettes, which I refuse to support. She already owes me 730PLN and hasn't repaid for over a year. When I offered to buy groceries instead, she stopped talking to me. She borrows from family and lenders but pays them back, making me feel unimportant. After confronting her, she accused me of thinking she's a failure. Am I wrong for refusing? How can I avoid worsening the conflict?	I (18F) work part-time at minimum wage and still live at home. My mom often borrows my car or money. I lent her $30 last week, but she complains whenever I ask for it back. Today, she messaged me saying a family member can't fly in for Thanksgiving without $100 for luggage and asked if I could spare it until next Friday. I told her no, and she called me a shitty person. Am I the asshole?	Very similar<br><br>Very similar<br><br>Very similar
Before my best friend (17F) got a boyfriend, we hung out all the time. But after she started dating, she began ignoring me to spend time with him and his friends. When I talked to her about it, she accused me of being jealous and left college to be with him, leaving me alone. As we grew distant, we argued more. I started spending time with other friends, and she got upset, accusing me of excluding her. She blocked me on everything and convinced our friends to drop me. I sent her a message saying I was tired of her behavior, only for her to call me a "shit friend." Am I wrong for feeling this way?	I (17M) had a close friendship with a girl (18F) who recently started getting close to a guy I dislike. We've texted every day for years and could always talk out our problems. Now, she's phasing me and other friends out to spend time with him. I told her it upset me, but she ignored my messages. On a recent free day, she acted like she didn't know me. When I sent a "we need to talk" text, she left me on read. Frustrated, I messaged, "I'm just gonna assume you didn't have time to answer so I don't get really pissed off." She blocked me without replying. Mutual friends think I'm overreacting, but it feels like I always make sacrifices in our friendship. I don't think I deserved to be blocked for wanting to talk. Am I the asshole for being upset she's willing to throw away our years of friendship for a guy she hardly knows?	Very similar<br><br>Very similar<br><br>Very similar
Last week, I (16M) visited home and spent time with my girlfriend (16F) for the first time in two months. The last night I could see her was Saturday, but she chose to go to a party instead. She invited me, but I declined because I don't enjoy parties. I told her I wanted to spend that night together since it would be a month before we saw each other again. She declined, saying the party had been planned for weeks, which she only informed me of the night before. I was upset because of our limited time together, while she was upset that I expected her to devote the whole week to me. Am I the asshole for wanting to spend time with her before I leave despite her plans?	My girlfriend (19F) and I (19F) haven't seen much of each other in 3 months due to her externship. She got back a few days ago, but we've both been busy with work and family. Today, she was supposed to get off work at 9pm. I planned to see the new Star Wars movie with my family and be home by 10pm, hoping we'd spend some time together before bed. After the movie, I saw she went out with a mutual friend instead. I don't mind her hanging out with others, but now she won't be home for hours, and I'll likely be asleep when she gets back. Am I the asshole for being upset she went out instead of coming home?	Very similar<br><br>Somewhat similar<br><br>Somewhat similar
I (19F) was in a relationship with a girl for a year and a half. Things were mostly fine, but by June, I felt my romantic feelings for her had faded. I decided to be honest and told her I wanted to remain friends. She blocked me but later unblocked and sent messages saying she hated me and felt abandoned. She accused me of making her suicidal and not caring about her during her rough patch. Both of us have struggled with depression, and her messages blaming me were affecting my own mental health. I stepped away to protect myself from the constant negativity. Am I selfish or an asshole for stepping away even though I initially wanted to be there for her?	I've been struggling with severe depression and anxiety after failing my exams and not getting into university. My girlfriend (19F) broke up with me after I told her she was the only thing stopping me from seriously hurting myself. We're part of a small friendship group that supported our relationship. When she went to university, she kissed someone else and said she felt "trapped." I had a mental breakdown during a visit, and she later ended things, saying I needed to help myself first. I told her I need to distance myself for my mental health, but this means avoiding our entire friend group. Am I the asshole for wanting to cut them out of my life until I feel better, even if it seems selfish?	Neither dissimilar nor similar<br><br>Somewhat similar<br><br>Somewhat dissimilar<br><br>Resolved as: Neither dissimilar nor similar

Continued on next page

Table S4 – continued from previous page

Submission 1	Submission 2	Evaluations
I'm (20F) socially anxious and often use sarcasm to cope. A friend (23M) I've had an on-off friends-with-benefits relationship with recently told me my sarcastic attitude was giving him anxiety. I decided to be vulnerable and open about my feelings, despite feeling uncomfortable. Mid-conversation, he blocked me on that platform. I felt ashamed and embarrassed, like I was coerced into vulnerability only to be hurt. A few days ago, he said he wanted to know if he upset me, so I messaged him on our usual platform, asking what happened. This led to an argument where he felt I was blaming him for being uncomfortable. Am I the asshole for being upset and wanting to discuss my feelings, or am I being selfish and playing the victim?	Me (M22 at the time) and my best friend (M26 at the time) haven't spoken for 3 years because I cut him off. In 2017, we were inseparable. When my mother was diagnosed with cancer, I withdrew to spend time with her. He was supportive. After my mother passed, he made an offensive mom joke within 3 months. When I told him it was not cool, he dismissed it as "just something dudes do." I sought an apology, but he insisted it meant nothing. I cut him off and distanced myself from our mutual friends. Recently, he lost his job due to COVID, and I'm considering recommending him for a job. Am I the asshole for cutting him off and distancing myself from others, and would it be too much to offer him a job interview now?	Very dissimilar<br><br>Somewhat dissimilar<br><br>Somewhat dissimilar
I'm 17M, and my stepsister is 18F. We've known each other for years but aren't close. She moved out recently, and our parents are going on vacation soon. They want her to come back and look after me. I don't mind her coming, but it's a 4-5 hour drive, and she just moved out. I feel bad making her do this just so our parents can force us to bond. I tried to convince my parents it's unnecessary, but they insist she comes so I won't be lonely. When I asked my stepsister, she said she doesn't want to but feels obligated because our parents pay for her phone and car. I feel guilty that she's being forced into this. AITA for trying to tell my parents that my stepsister looking after me is unnecessary?	I (19F) live with my boyfriend, best friend, and her girlfriend. We split the rent. Recently, my stepsister got accepted to a college nearby and wants to move in with us. My dad and his wife offered to pay her share of the rent, thinking it would help us bond, but I said no. My dad's wife called, saying it was wrong and cruel to turn her away and that her daughter wanted to bond as adults since we never had a sibling relationship growing up. I told her I didn't want to live with my stepsister and wanted to keep my current living situation. AITA for saying no without consideration? My dad and his wife think I'm being unfair, but I don't see her as family or a friend, so I don't feel bad.	Somewhat dissimilar<br><br>Somewhat dissimilar<br><br>Somewhat dissimilar
